# Pneumococcal colonization dynamics among young children with and without respiratory symptoms during the first year of the SARS-CoV-2 pandemic

**DOI:** 10.1371/journal.pone.0327046

**Published:** 2025-06-26

**Authors:** Liset Olarte, Brian Lee, Dithi Banerjee, Douglas S. Swanson, Christopher J. Harrison, Rangaraj Selvarangan

**Affiliations:** 1 University of Missouri–Kansas City School of Medicine, Missouri, United States of America; 2 Department of Pediatrics, Children’s Mercy, Kansas City, Missouri, United States of America; 3 Department of Pathology and Laboratory Medicine, Children’s Mercy, Kansas City, Missouri, United States of America; Indian Institute of Technology BHU Varanasi, INDIA

## Abstract

**Background:**

Non-pharmaceutical interventions to mitigate the spread of SARS-CoV-2 were implemented across the United States in 2020. These public health measures might influence pneumococcal colonization in younger children and their subsequent risk of invasive pneumococcal disease. Our objective was to evaluate pneumococcal colonization rates in children ≤ 5 years of age with and without respiratory symptoms during the first 12 months of SARS-CoV-2 pandemic (April 2020 – March 2021).

**Study design:**

This is a retrospective cross-sectional study evaluating pneumococcal colonization rates, density and serotype distribution across three study periods (April-July [Period 1], August-November [Period 2] and December-March [Period 3]) after implementation of non-pharmaceutical interventions in Kansas City, Missouri. Children aged ≤5 years with and without respiratory symptoms seeking care at Children’s Mercy Kansas City hospital system with a residual standard of care nasal mid-turbinate sample were included. The odds of pneumococcal colonization across study periods were calculated using multivariable logistic regression.

**Results:**

A total of 311 children met inclusion criteria (126 symptomatic and 185 asymptomatic). The overall pneumococcal colonization (23% vs. 13%, p = 0.03) and SARS-CoV-2 positivity (34.1% vs. 17.8%, p = 0.001) rates were higher in symptomatic children than in asymptomatic children. The odds of pneumococcal colonization of symptomatic (Period 2: OR 1.09; 95% CI 0.33–3.64, and Period 3: OR 0.46; 95% CI 0.13–1.59) and asymptomatic (Period 2: OR 0.55; 95% CI 0.18–1.7, and Period 3: OR 0.37; 95% CI 0.11–1.2) children did not statistically differ across study periods. Pneumococcal colonization density was also not different across study periods among study groups. Overall, non-PCV15, non-PCV20 serotypes were the most frequently identified serotypes (56.8%).

**Conclusions:**

Pneumococcal colonization rates and density did not significantly change across study periods as adherence to non-pharmaceutical interventions gradually relaxed during the first year of the SARS-CoV-2 pandemic.

## Introduction

Nasopharyngeal colonization with *Streptococcus pneumoniae* plays a pivotal role in the development of mucosal and invasive pneumococcal diseases (IPD). Children ≤5 years of age have the highest rates of pneumococcal colonization and contribute to horizontal dissemination of pneumococcus within communities. Daycare and school attendance, and other crowding conditions are considered risk factors for pneumococcal colonization [1]. Presence of a respiratory virus even in the absence of symptoms has been suggested to increase pneumococcal colonization rates and facilitate pneumococcal transmission and disease [2,3].

The surge of SARS-CoV-2 cases observed in March 2020 in the United States prompted the implementation of non-pharmaceutical interventions (NPI) (social distancing, frequent handwashing, wearing masks in public, and shelter-in-place orders) to reduce the spread of SARS-CoV-2 [4]. These interventions not only reduced the transmission of SARS-CoV-2 and other respiratory viruses, but also reduced the incidence of IPD and other bacterial infections [5]. In 2020, among 26 countries in six continents, the incidence of IPD decreased by 82% at 8 weeks following NPI implementation compared with corresponding data from 2018 and 2019 [5]. The causes of the altered dynamics of respiratory infections during the first years of the SARS-CoV-2 pandemic are incompletely understood. However, the changes in human behavior resulting from the implementation of NPI appeared to be a substantial factor that in turn lead to alterations in nasopharyngeal microbiota, changes in nasopharyngeal colonization rates and density, resurgence of endemic viruses outside their typical seasons, waning immunity, increase severity of rebounded viral and bacterial infections, etc. It has been hypothesized that the decrease in IPD incidence was secondary to an interruption in the transmission of *S. pneumoniae* leading to decrease rates of pneumococcal colonization. Studies from France, Belgium and Israel reported no differences in pneumococcal colonization in children before and during the SARS-CoV-2 pandemic, while a study from Serbia reported increased pneumococcal colonization during the pandemic [6–9]. However, there is little data on the effect of NPI on pneumococcal colonization in children in the United States. We hypothesized that pneumococcal colonization rates in children ≤ 5 years of age would have decreased with the implementation of NPI and then would gradually increase due to two factors: relaxation of NPI plus onset of the winter season in the Kansas City region. We evaluated serotype-specific pneumococcal colonization dynamics in children with and without respiratory symptoms during the first 12 months of SARS-CoV-2 pandemic.

## Materials and methods

### Study design

This is a retrospective cross-sectional study evaluating variations in pneumococcal colonization prevalence and serotype distribution among children aged ≤5 years with and without respiratory symptoms, and seeking care at Children’s Mercy - Kansas City (CMKC) hospital system during the first 12 months of SARS-CoV-2 pandemic (April 1, 2020 to March 31, 2021). The CMKC population coverage area and demographics are detailed in S1 Appendix in S1 File. The study period was divided in three 4-month periods: April/May/June/July (Period 1, spring-summer), August/September/October/November (Period 2, summer-fall) and December/January/February/March (Period 3, winter-spring). Period 1 represents the time of strict recommendations for NPI, e.g., social distancing, masking and shelter-in-place orders to reduce the spread of SARS-CoV-2. Period 2 represents the time when NPI were at least partially lifted in our region, with cities reopening to activities and children being back in daycare and school. Period 3 represents winter season with maintenance of partial NPI. NPI implementation and relaxation dates are listed in S2 Appendix in S1 File. This retrospective study was approved by the Institutional Review Board at CMKC with a waiver of consent and HIPAA authorization (#STUDY00001775) because research involved no more than minimal risk to the subjects.

### Study population

Children aged ≤5 years who had a residual sample remaining from a nasal mid-turbinate specimen obtained for viral testing as part of routine care were included in this study. The asymptomatic group consisted of children without respiratory symptoms, but requiring SARS-CoV-2 testing. Two subgroups made up the asymptomatic group: (1) children requiring SARS-CoV-2 polymerase chain reaction (PCR) screening 72 hours prior to a scheduled surgery or a sedated procedure (pre-procedure) and exhibiting no respiratory symptoms (rhinorrhea, nasal congestion, cough, sore throat, earache, fever, respiratory distress/shortness of breath), and (2) children exposed to SARS-CoV-2 but exhibiting no respiratory symptoms as determined by the ordering provider. The symptomatic group consisted of children with respiratory symptoms that sought care at CMKC and required SARS-CoV-2 PCR and/or other viral PCR testing (BioFire® FilmArray® Respiratory Panel or Cepheid Xpert® Xpress Flu/RSV) as recommended per treating provider. SARS-CoV-2 testing required the ordering provider to select an indication for testing; the main categories included: asymptomatic exposed, asymptomatic pre-procedure, and symptomatic. Eligible children were identified by performing an electronic health record (EHR) query. Convenience sampling was used and stratified within the asymptomatic and symptomatic groups for each study period. Samples were obtained from ten different CMKC facilities across Kansas City (S1 Appendix in S1 File). This approach helped diversify the study population, reducing the risk that the convenience sample would represent only a narrow subset of the population. If subjects had multiple encounters generating a residual specimen during the study period, only the first encounter was considered for inclusion. Subject identification and data collection started on 01/11/2021.

### Data collection

Demographic and clinical data such as age, sex, smoking exposure, pneumococcal immunization, complex chronic conditions, and viral testing result were obtained from EHR. The CMKC Integrated Care Solutions Data Repository (network that coordinates the medical care of patients enrolled in commercial insurance and Medicaid plans) was queried to obtain pneumococcal immunization and antibiotic use (within 4 weeks prior to sample collection), which could impact colonization results. Additional pneumococcal immunization information was also obtained from the Kansas and Missouri immunization registries. As reference, the coverage of ≥ 3 PCV13 doses by age 35 months in Missouri and Kansas was 88.6% (95% CI: 82.9–93.1%) and 92.7% (95% CI: 88–96.1%), respectively, in 2019 [10].

### Pneumococcal colonization and molecular serotyping

Samples were collected using flocked swabs. Each swab was placed into a 3 ml tube of Universal Transport Medium and underwent viral testing; the sample residual was stored at –80⁰C. De-identified samples were used for the following research procedures. Deoxyribonucleic acid (DNA) was extracted using QIAGEN DNeasy (Germantown, Maryland, USA) automated extraction system and human-specific glyceraldehyde 3-phosphate dehydrogenase (GAPDH) real-time PCR was used to evaluate quality of samples. Real-time PCR assay targeting *lytA* gene was used for *S. pneumoniae* detection with a cycle threshold value ≤35 considered positive [11]. Positive samples for *S. pneumoniae* underwent sequential multiplex serotype-specific PCR testing which detects 40 pneumococcal serotype/serogroups (1, 2, 3, 4, 5, 6A/6B/6C/6D, 6C/6D, 7C/7B/40, 7F/7A, 8, 9N/9L, 9V/9A, 10A, 10F/10C/33C, 11A/11D, 12F/12A/12B/44/46, 13, 14, 15A/15F, 15B/15C, 16F, 17F, 18C/18F/18B/18A, 19A, 19F, 20, 21, 22F/22A, 23A, 23B, 23F, 24F/24A/24B, 31, 33F/33A/37, 34, 35A/35C/42, 35B, 35F/47F, 38/25F/25A, 39) and each reaction includes the internal control gene *cpsA* [12]. Amplified PCR products were analyzed by gel electrophoresis. To calculate the colonization density, a standard curve of *lytA* was generated with known DNA concentrations from control strains, plotted against the cycle threshold values to calculate CFU/ml and then transform to log10.

### Sample size

The pneumococcal colonization rate in children <5 years in the United States is ~ 30% [13,14]. Higher colonization rates (40−50%) have been reported in children with respiratory infections [3,15]. We hypothesized pneumococcal colonization rates during Period 1 would be lower than these previously reported rates due to interventions implemented to reduce the spread of SARS-CoV-2, followed by a gradual increase of 10% for asymptomatic group and 15% for symptomatic group in colonization rates from period to period. We used Cochran-Armitage trend test to calculate the sample size assuming the above trend of pneumococcal colonization rates, 80% power, and equal sample size per period. The total calculated sample size for asymptomatic group was 186 children (62 per period) and for symptomatic group was 126 children (42 per period).

### Statistical analysis

Pearson’s chi-square and Fisher’s exact test were used for when examining bivariate associations between categorical variables. The overall proportion of children with pneumococcal colonization and vaccine serotypes were compared across study periods using the Cochran-Armitage test for trend. Pneumococcal colonization density was compared across study periods using the Kruskal-Wallis test and Wilcoxon Rank-sum test, where appropriate. Nearly all variables included in this analytic framework were categorical with the exception of colonization density. The colonization density variable exhibited a slight positive skewness, though we did not consider it substantial enough to necessitate any data transformation or outlier exclusion. Given the relatively small number of positive specimens, we presented non-parametric comparisons.

We performed multivariable logistic regression modeling to examine the relationship between pneumococcal colonization and study periods. Variables included in the logistic regression models were identified *a priori* and included the following potential confounders: sex, exposure to tobacco smoke, complex chronic conditions, pneumococcal immunization, prior antibiotic use, and SARS-CoV-2 positive results. This list was further refined based on the strength of associations observed during the bivariate comparisons. The regression models were built incrementally, which allowed for an assessment for any change in coefficient directionality, significant changes in adjusted coefficients, and significant changes in standard errors. More robust multicollinearity assessments were not completed. Analyses were stratified into asymptomatic and symptomatic groups. No imputation methods were performed as missing data were limited to two variables: SARS-CoV-2 positivity (<1%) and PCV-13 vaccination status (<8%). Missing SARS-CoV-2 status applied only to 3 symptomatic participants. Missing PCV-13 vaccination records were handled by categorizing immunization into three groups: ≥ 3 doses, 0–2 doses, and no vaccine record. We further conducted an analysis excluding the participants without vaccine records, and the results remained consistent, indicating that missingness did not influence our results. Analyses were completed using Stata (StataCorp. *Stata Statistical Software: Release 17*. College Station, TX).

## Results

Of the 312 samples, one (from the asymptomatic group) was GADPH negative and excluded from the analysis. Selected characteristics of the overall asymptomatic and symptomatic groups are presented in Table 1. All 185 asymptomatic children had SARS-CoV-2 testing performed; 152 (82.2%) prior to a sedated procedure (categories of procedure listed in S1 Table in S1 File) and 33 (17.8%) because they were SARS-CoV-2 exposed children exhibiting no symptoms. A significant proportion of asymptomatic children requiring a sedated procedure were younger than 2 years of age, male and had a complex chronic condition when compared to asymptomatic children exposed to SARS-CoV-2 (S2 Table in S1 File). SARS-CoV-2 testing was performed in 123/126 (97.6%) symptomatic children. Three symptomatic children were not tested for SARS-CoV-2, but they were included because they had a nasal mid-turbinate specimen for respiratory panel testing. Complex chronic conditions [16] (S3 Table in S1 File) were more common in the asymptomatic group than the symptomatic group (40.5% vs 23.8%, p = 0.002). For the symptomatic group, the most common symptoms documented were fever (65.9%), cough (61.9%), nasal congestion (53.2%), rhinorrhea (28.6%), vomiting (17.5%), shortness of breath (16.7%), diarrhea (13.5%), otalgia (7.9%), sore throat (6.3%), and headache (2.4%). Pneumococcal immunization data were not available for 24 children (7.7%). Among children with confirmed pneumococcal immunization data, 205/287 (71.4%) received ≥ 3 doses of 13-valent pneumococcal conjugate vaccine (PCV13) (120/168 asymptomatic and 85/119 symptomatic children). The overall SARS-CoV-2 positivity rates for asymptomatic and symptomatic children were 17.8% and 34.1% (p = 0.001), respectively. Within the asymptomatic group, the SARS-CoV-2 positivity rate among children exposed to SARS-CoV-2 was higher than among children requiring a sedated procedure (39.4% vs. 13.4%, p = 0.0004). However, pneumococcal colonization rates were similar in the two groups (S2 Table in S1 File). The overall pneumococcal colonization rates for asymptomatic and symptomatic children were 13.5% and 23% (p = 0.03), respectively. SARS-CoV-2 positivity rate was similar between colonized and non-colonized children (22.2% vs. 25.2%, p = 0.65). Of the 54 samples positive for pneumococcal colonization, 12 (22.2%) were also positive for SARS-CoV-2, 2 (3.7%) for rhinovirus/enterovirus, and 1 (1.8%) for coronavirus NL63.

**Table 1:** Selected characteristics of asymptomatic and symptomatic groups.

		Asymptomatic (N = 185)	Symptomatic (N = 126)	p value
Age				0.043
	<2 years	93 (50.3%)	78 (61.9%)	
	2-5 years	92 (49.7%)	48 (38.1%)	
Sex				0.441
	Female	77 (41.6%)	58 (46.0%)	
	Male	108 (58.4%)	68 (54.0%)	
Race/ethnicity				<0.001
	Hispanic	23 (12.4%)	32 (25.4%)	
	Black (non-Hispanic)	19 (10.3%)	32 (25.4%)	
	White (non-Hispanic)	126 (68.1%)	54 (42.9%)	
	Other/unknown	17 (9.2%)	8 (6.3%)	
Complex chronic condition				0.002
	No	110 (59.5%)	96 (76.2%)	
	Yes	75 (40.5%)	30 (23.8%)	
Smoking exposure				0.407
	Yes	24 (13.0%)	12 (9.5%)	
	No	154 (83.2%)	106 (84.1%)	
	Unknown	7 (3.8%)	8 (6.3%)	
Number of PCV13 doses				0.505
	0	17 (9.2%)	14 (11.1%)	
	1	22 (11.9%)	10 (7.9%)	
	2	9 (4.9%)	10 (7.9%)	
	3	32 (17.3%)	26 (20.6%)	
	4	88 (47.6%)	59 (46.8%)	
	Unknown	17 (9.2%)	7 (5.6%)	
Number of PPSV23 doses				0.335
	0	167 (90.3%)	117 (92.8%)	
	1	1 (0.5%)	2 (1.6%)	
	Unknown	17 (9.2%)	7 (5.6%)	
Prior antibiotic use				0.307
	No	184 (99.5%)	123 (97.6%)	
	Yes	1 (0.5%)	3 (2.4%)	
Location of sample collection				0.471
	Outpatient setting	136 (73.5%)	98 (77.8%)	
	Inpatient setting	49 (26.5%)	28 (22.2%)	
Respiratory panel testing				<0.001
	Not tested	173 (93.5%)	100 (79.4%)	
	Negative	11 (5.9%)	18 (14.3%)	
	Positive^a^	1 (0.5%)	8 (6.3%)	
SARS-CoV-2 PCR test^b^				0.001
	Negative	152 (82.2%)	80 (65.0%)	
	Positive	33 (17.8%)	43 (35.0%)	
Pneumococcal colonization				0.03
	Negative	160 (86.5%)	97 (77.0%)	
	Positive	25 (13.5%)	29 (23.0%)	

^a^Rhinovirus/enterovirus (n = 5), coronavirus NL63 (n = 2), respiratory syncytial virus (n = 1), and adenovirus (n = 1).

^b^Three patients in the symptomatic group did not have a SARS-CoV-2 test done; thus the denominator used was 123. One of these three patients was positive for rhinovirus/enterovirus. PPSV23, 23-valent pneumococcal polysaccharide vaccine

**Fig 1:**
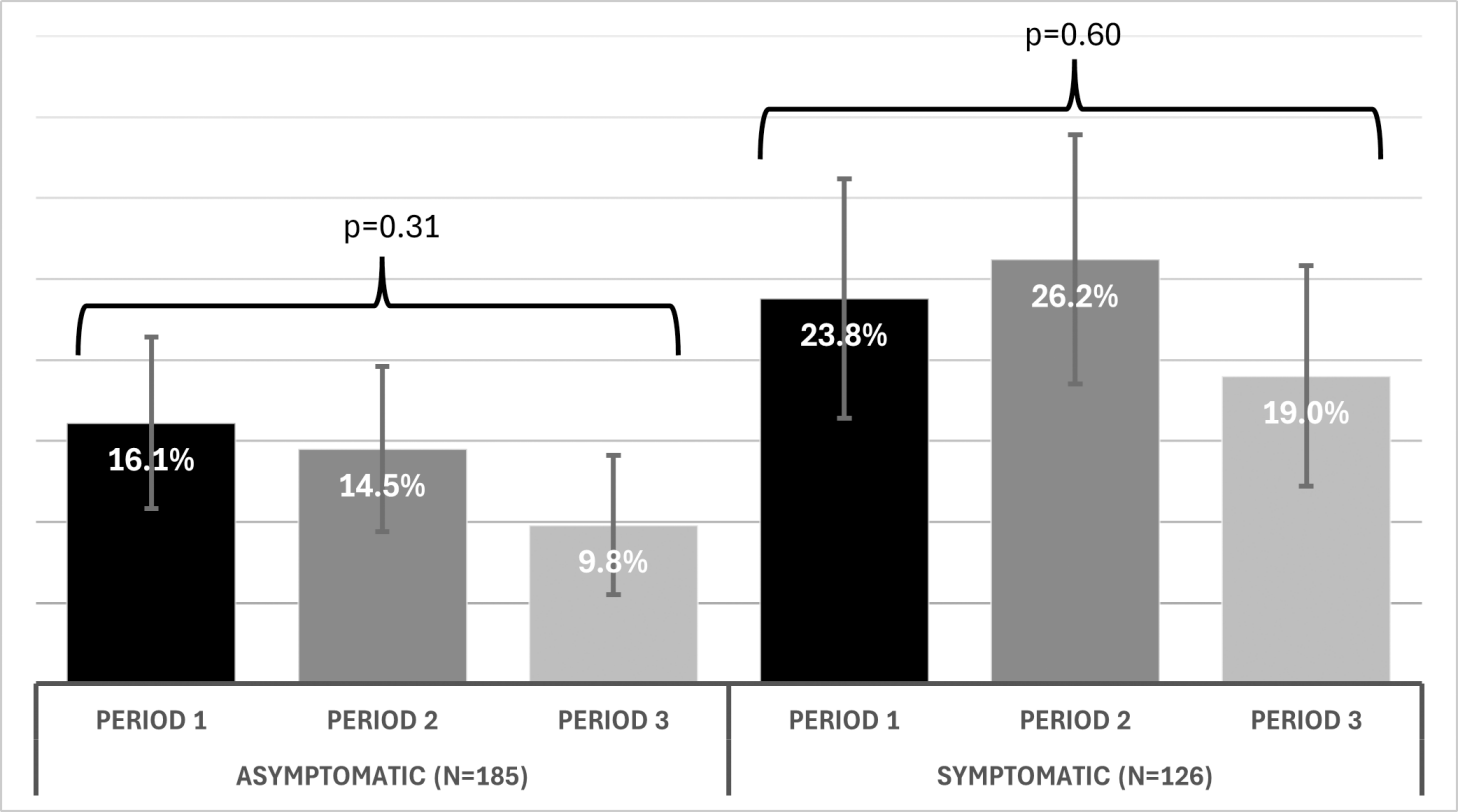
Pneumococcal colonization rates in asymptomatic and symptomatic patients by study periods.

The pneumococcal colonization rate did not change across study periods (Fig 1). The odds of colonization of asymptomatic children were similar during Period 2 (odds ratio [OR] 0.55; 95% confidence interval [CI] 0.18–1.7) and Period 3 (OR 0.37; 95% CI 0.11–1.2), using Period 1 as reference and after adjusting for age, sex, SARS-CoV-2 results, presence of a complex chronic condition, and ≥ 3 doses of PCV13. When compared with Period 1, the odds of colonization of symptomatic children were also similar for Period 2 (OR 1.09; 95% CI 0.33–3.64) and Period 3 (OR 0.46; 95% CI 0.13–1.59).

Pneumococcal serotype was confirmed in 44/54 (81.5%) positive samples. The most common serotypes were 15A/15F (n = 8), 11A/11D (n = 6), 35B (n = 5), 15B/15C (n = 4), 19F (n = 3), 23A (n = 3), and 23B (n = 3). Seven of 44 (15.9%) serotypes were PCV13 serotypes (19F [n = 3], 19A [n = 2] and 3 [n = 2]). Six of seven (85.7%) patients colonized with a PCV13 serotype had received ≥ 3 doses of PCV13. Two isolates (4.5%) were among the serotypes unique to PCV15 and 10 isolates (22.7%) among the serotypes unique to PCV20. Twenty-five isolates (56.8%) were serotypes that would not be covered by PCV13, PCV15 or PCV20.

**Fig 2:**
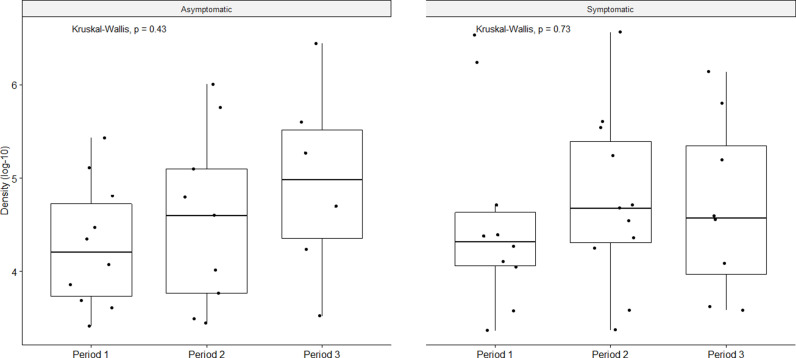
Pneumococcal colonization density across the three study periods for the asymptomatic and symptomatic groups.

**Fig 3:**
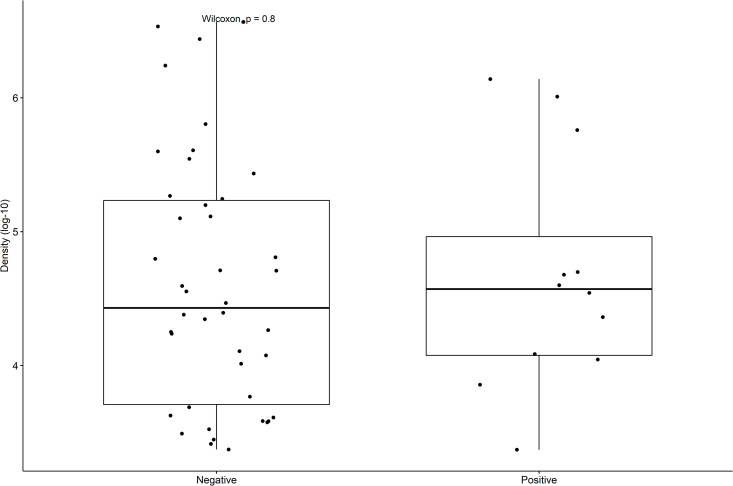
Pneumococcal colonization density by SARS-CoV-2 results.

**Fig 4:**
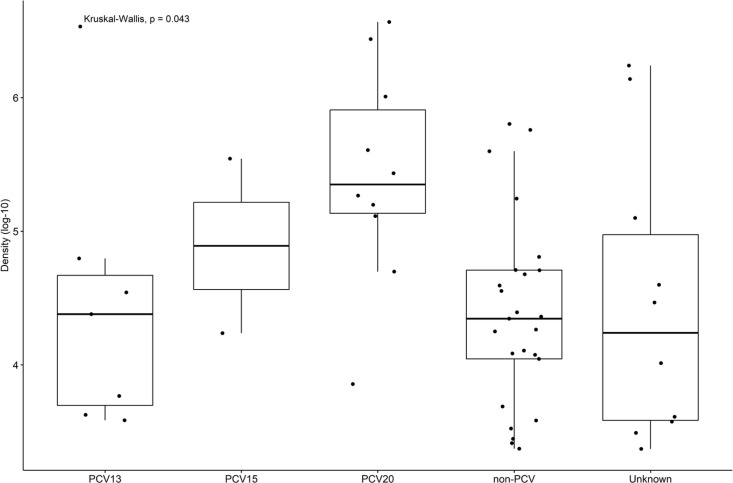
Pneumococcal colonization density by vaccine serotype categories. PCV15 category includes serotypes unique to PCV15 (non-PCV13), PCV20 category includes serotypes unique to PCV20 (non-PCV13 and non-PCV15), non-PCV category includes serotypes not covered by PCV13, PCV15 or PCV20, and unknown represents the samples without a confirmed serotype. A post-hoc analysis was completed following the Kruskal-Wallis test, with p-values adjusted for multiple comparisons. A significant difference in colonization density was observed between PCV20 and non-PCV categories (p = 0.017).

Pneumococcal density was not different across study periods for the asymptomatic and symptomatic groups (Fig 2). Colonization density was also not impacted by SARS-CoV-2 positivity (Fig 3). The highest colonization density was observed among unique PCV20 serotypes (Fig 4).

## Discussion

In our study, pneumococcal colonization rates and density did not significantly change across study periods despite NPI gradually relaxing and winter season starting over the first year of the SARS-CoV-2 pandemic. However, our results may have been impacted by our relatively small sample size and the absence of a pre-pandemic comparison group. Pneumococcal colonization (23% vs. 13%, p = 0.03) and SARS-CoV-2 positivity (34.1% vs. 17.8%, p = 0.001) rates were higher for children in the symptomatic group than in children exhibiting no respiratory symptoms, which is consistent with previous studies [3,15]. As might be expected, the most common detected serotypes were serotypes not covered by PCV13, PCV15 or PCV20 (56.8%). Only 7 children were colonized with PCV13 serotypes, but 6 had received ≥ 3 doses of PCV13. Interestingly, colonization density for PCV13 serotypes was lower compared with higher-valency vaccine serotypes. This was also not unexpected because PCV15 and PCV20 were not in use prior to or during the study period.

Our study did not include a pre-pandemic control group, and pneumococcal colonization rates for Kansas City children under 5 years of age before 2020 are unavailable for comparison. The most comparable pre-pandemic data comes from a study conducted in St. Louis, Missouri, involving young mostly asymptomatic children who required sedation for procedures or minor surgeries from 2013 to 2016 [17]. This study found a 30.8% and 28.9% pneumococcal colonization prevalence among children 0–2 years old and >2–6 years old, respectively. Using these data as a pre-pandemic comparison, the pneumococcal colonization rates observed in Period 1 of our study were substantially lower for the asymptomatic group (16.1%) than the symptomatic group (23.8%); lower rates in the asymptomatic group were present in each study period. It is noteworthy that detected colonization rates in our study were lower despite pneumococcal identification using PCR-based methods, which are more sensitive that the culture-based methods used in the St. Louis study. However, without local rates from the months immediately preceding the implementation of the shelter-at-home order, it is challenging to contextualize our observed rates from Period 1.

In 2020, IPD rates were historically low in the United States and worldwide [5,18–20]. The greatest reduction in IPD cases occurred during the second and third quarters of 2020 coinciding with total lockdown [19,21]. This was thought to be secondary to a decrease in pneumococcal colonization due to stringent NPI. However, studies from France, Israel, Belgium and South Africa reported no change in pneumococcal colonization rates in young children before and during the pandemic, proposing that the temporary decline in IPD was associated with interruption of the transmission of respiratory viruses [6–8,22].

To our knowledge, the only United States study evaluating pneumococcal colonization in children during the pandemic included adult data in the analysis. This was a community-based household surveillance study evaluating the association of respiratory viruses and pneumococcal semiquantitative colonization density from November 2019 to June 2021 in Seattle, Washington [23]. Their overall (children plus adult) pneumococcal colonization rate from November 2019 to March 2020 was 12%; and declined to 6% and 5% in the March-December 2020 and January-June 2021 periods, respectively [23]. Their lower rates may have been due to the fact that colonization rates vary and tend to decrease with age.

Our study evaluated pneumococcal colonization exclusively in children ≤ 5 years of age in three periods after NPI implementation. The odds of pneumococcal colonization remained relatively stable across study periods for symptomatic and asymptomatic children. A slightly lower pneumococcal colonization rate in Period 3 (December 2020 – March 2021) was not statistically lower compared to the other two periods for either study groups. We anticipated a higher colonization rate in Period 3 compared to Period 1 based on the previous knowledge that pneumococcal colonization rates and IPD increase during winter when seasonal respiratory viruses circulate [24,25]. That said, influenza, respiratory syncytial virus, and human metapneumovirus circulation was markedly low during the winter of 2020–2021 [26], which may have contributed to an overall unchanged colonization rate and the absence of the usual seasonal-virus-induced uptick in pneumococcal colonization in the third period of our study. Other studies also showed a somewhat lower colonization rate towards the end of 2020; however, direct comparisons with our study are challenging due to differences in pneumococcal identification techniques, age groups, overall timeframes, and specific timing of NPI [7,23]. In Israel, pneumococcal colonization rates from June 2020 to February 2021 were not significantly different from previous years, except for lower rates in the October-December 2020 period [7]. In contrast, Belgian data showed high colonization rates (67.4%) during the winter 2020–2021 (November to March) in children <3 years of age, a rate comparable to previous winters; however, these investigators did not assess colonization rates during early pandemic months [8].

In Serbia, a significant increase in pneumococcal colonization was noted from February-March 2020 to September-November 2020 (24.9% vs. 32.1% p = 0.0085) and to April-June 2021 (24.9% vs. 37.7%, p < 0.0001) [9]. However, this study design differed from our study, included only 2–5 year old children and combined data from symptomatic and asymptomatic children. Further, the largest Serbian increase was not detected until April-June 2021, after the COVID-19 rates in children had plunged. One could speculate that our study timeframe stopped short of a potential increase in colonization as was seen in Serbia. Children attending daycare were also over represented among the colonized group, about 84.6% of their participants [9]. We did not have access to daycare attendance data, which is a known risk factor for transmission of upper respiratory tract pathogens. However, during the initial months of NPI, daycare facilities were closed with few exceptions. As a result, daycare attendance during Period 1 was likely lower than in subsequent periods when daycare centers were allowed to reopen. That said, we were unable to evaluate whether the unchanged colonization rate across study periods was related to limited daycare attendance, despite the relaxation of NPIs in Periods 2 and 3.

Another confounding factor may have been the fact that single sample snapshot surveillance colonization rates necessarily detect colonization that has been present for varying lengths of time. Colonization has been reported to last a median of 60 days, with some persisting for 12 months [27]. So, colonization detected later during period 1 (three months into onset of the pandemic) may have persisted into the second period, so that rates did not decrease as much as we anticipated. It is also possible that strict NPI may have not been in place long enough to see a significant impact on pneumococcal colonization rates. However, some families may have remained more cautious about the level of exposures in preschool age children despite gradual relaxation of NPI, so that recolonization was less likely.

The few studies reporting pneumococcal serotype distributions revealed no difference from previous years [6,7,9]. We reported a predominance of non-PCV15, non-PCV20 serotypes with 15A and 35B among the most common serotypes; our data are consistent with a United States colonization study conducted from 2021 to 2023 [28]. Some of these non-PCV15, non-PCV20 serotypes (serotypes 35B, 15A, 23A, 23B) are also among the most common serotypes causing acute otitis media and IPD in children in the United States [29]. Although these relevant serotypes are not included in current pediatric PCVs, they are included in a new 21-valent PCV recently approved for use in the adult population and under evaluation for pediatric use.

Regarding overall pneumococcal colonization density, we did not observe a difference across the three study periods in either study group. This is consistent with findings in Seattle and Israel where colonization density did not change after NPI implementation [7,23]. Yet, studies from South Africa and Vietnam reported a reduction in pneumococcal colonization density even when colonization rates remain unchanged in those areas; suggesting that the IPD decline may have been secondary to a decline in *S. pneumoniae* density after NPI implementation [22,30].

We observed a trend toward higher density among serotypes in the higher valency vaccines. Indeed, pneumococcal colonization density in our study among PCV13 serotypes was lower than that among serotypes unique to PCV15 and PCV20. This lower density may be related to a protective effect of PCV13 vaccination as most children (71.4%) included in our study had received ≥ 3 doses of PCV13. However, studies have reported inconsistent results regarding the effect of PCVs on pneumococcal nasopharyngeal density in children [31]. The slightly higher colonization density observed in the asymptomatic group in Period 3 seems to be driven by serotypes unique to PCV20, but our numbers are too small in the subcategories to draw any conclusions.

Pneumococcal colonization density can be influenced by viral co-detection, which our study was not designed to thoroughly evaluate. Almost all children were tested for SARS-CoV-2 (308/311), but a considerable proportion were not evaluated for other viruses, potentially impacting density results. We found a 3.9% (12/308) co-detection of *S. pneumoniae* and SARS-CoV-2, and no difference in colonization density based on SARS-CoV-2 positive results. This finding may suggest independent pathogenic dynamics between *S. pneumoniae* and SARS-CoV-2, a distinct host-pathogen interaction, or methodological factors such as the timing of sample collection during the illness. Studies from Seattle and Belgium did not identify any *S. pneumoniae* and SARS-CoV-2 co-detection; however, co-detection with other viruses was reported in Seattle [8,23]. Our somewhat higher virus co-detection may indicate that children included in our study had more social interactions or healthcare related exposures due to our patients’ relatively high rate of complex chronic conditions which likely required more frequent medical attention, particularly in the asymptomatic group. Or it may indicate a less than complete adherence to NPI.

The strengths of our study include the parallel evaluation of serotype-specific pneumococcal colonization in two groups of children based on the presence or absence of respiratory symptoms during the SARS-CoV-2 pandemic, the availability of pneumococcal immunization data for most children (92.3%) and the use of quantitative methods to determine S. pneumoniae density. Nonetheless, the findings in this study are subject to some limitations. First, the number of subjects included in each study period may not have been large enough to detect a significant difference. Second, we do not have data on adherence to NPI or social behavior practices of the children or their household contacts due to the retrospective design of the study. It is possible that some families were not able to strictly adhere to NPI at the beginning of the pandemic (e.g., essential workers); while other families decided to continue limiting their social interactions even when schools and daycares reopened. Variation in NPI adherence and care-seeking behaviors could have introduced potential confounders. Third, we compared pneumococcal colonization rates during discrete time periods across the first year of the SARS-CoV-2 pandemic after NPI implementation; however, the absence of pre-pandemic baseline pneumococcal colonization rate data limits the trend analysis of our results. Fourth, a large percentage of children in this study were not evaluated for respiratory viruses other than SARS-CoV-2. Thus, undetected respiratory virus co-infection could have impacted pneumococcal colonization density results, particularly for the asymptomatic group. In addition, our study timeframe did not continue to the time when other viral infection rates increased. Fifth, this was a cross-sectional study; therefore, repeat samples from each child over time were not obtained to evaluate whether NPI impacted the acquisition of pneumococcus or new pneumococcal serotypes. Lastly, this is a single-center study and we used a convenience sample, both of which limit the external validity of our results, as they cannot be readily generalized to other areas where NPI and PCV coverage may differ. Convenience sampling may have also contributed to over-representation of children with complex chronic conditions compared to other studies.

In conclusion, we did not detect significant decrease in pneumococcal colonization after NPI was in place for several months, nor did we detect an increase when NPI was relaxed and winter season effects could have been expected. The anticipated changes in overall pneumococcal colonization rates and density among children with and without respiratory symptoms were not observed despite shifting strictness of recommended NPI during the first year of the SARS-CoV-2 pandemic. Even though our results may have been impacted by a relatively small sample size in each study period, our results reflect those of studies using national surveillance systems which also did not report significant changes in pneumococcal colonization. Integrating more completely documented human behavioral monitoring in future prospective multicenter studies is of utmost importance for thoroughly understanding the impact of NPI on the complex pneumococcal colonization dynamics, as well as the viral-bacterial interactions in pneumococcal transmission and disease pathogenesis.

## Supporting information

S1 File S1 Appendix. Children’s Mercy Kansas City population coverage area and demographics. S2 Appendix. Dates of community mitigation measures implemented in the Kansas City Metro Area. S1 Table. Procedure categories for which asymptomatic group required SARS-CoV-2 testing, by pneumococcal colonization status. S2 Table. Characteristics of asymptomatic participants. S3 Table. Complex chronic condition categories identified among patients in the asymptomatic and symptomatic groups. S1 Data. Minimal anonymized dataset.
(ZIP)
