# Supplementary material for: Pneumococcal colonization dynamics among young children with and without respiratory symptoms during the first year of the SARS-CoV-2 pandemic
Source: PLoS One. 2025 Jun 26;20(6):e0327046. doi: 10.1371/journal.pone.0327046 (PMC12200735; doi:10.1371/journal.pone.0327046)
Supplement: S1 File — S2 Appendix. Dates of community mitigation measures implemented in the Kansas City Metro Area. S1 Table. Procedure categories for which asymptomatic group required SARS-CoV-2 testing, by pneumococcal colonization status. S2 Table. Characteristics of asymptomatic participants. S3 Table. Complex chronic condition categories identified among patients in the asymptomatic and symptomatic groups. S1 Data. Minimal anonymized dataset. (ZIP) [file pone.0327046.s001.zip › S1 Appendix.docx]

**S1 Appendix**. Children’s Mercy Kansas City population coverage area and demographics

- Children's Mercy Kansas City (CMKC) is a free-standing, nonprofit 366 bed academic pediatric health system that provides comprehensive primary and tertiary specialty care to children from Missouri and Kansas, as well as neighboring states. CMKC Main campus is located in Kansas City, Missouri and CMKC South Campus in Overland Park, Kansas.
- CMKC is the region’s pediatric provider choice as we have over 85% market share among pediatric medical/surgical patients in the 6-County KC Metro Service Area.
- CMKC's primary service area consists of 18 counties, nine each in the states of Missouri and Kansas.
- Outpatient facilities: During FY 2019, there were 332,133 outpatient specialty visits and 189,076 emergency/urgent care visits
  - Ambulatory pediatric care is predominantly delivered at:
    - Main campus ambulatory building
    - South Campus specialty clinics
    - Children’s Mercy Northland clinic (approx. 14 miles from the main campus)
    - Children’s Mercy Clinics on Broadway (approx. 2 miles from the main campus)
  - Urgent and emergent care is provided in the Emergency Department facilities located in the Main and South Campuses; and the three suburban urgent care centers (2 in Missouri and 1 in Kansas).
- Inpatient admissions: During FY 2019, there were 16,021 inpatient admissions with approximately 89,718 total patient-days, including 24,419 NICU patient days and 14,381 PICU patient-days. In addition, there were 20,262 total surgical procedures.
- The demographics of the population served by CMKC (from fiscal year 2020) are as follows:

| **Age Range** | **Total** |  | **Gender** | **Total** |  | **Race/ethnicity** | **Total** |
| --- | --- | --- | --- | --- | --- | --- | --- |
| Infant (<1 year old) | 12.1% |  | F | 48.7% |  | American Indian or Alaska Native | 0.3% |
| 1-4 y | 27.6% |  | M | 51.3% |  | Asian | 2.3% |
| 10-14 y | 23.2% |  | **Total** | **100%** |  | Hispanic | 12.1% |
| 15-17 y | 12.5% |  |  |  |  | Multiracial | 5.9% |
| 18+ y | 4.6% |  |  |  |  | Native Hawaiian or Other Pacific Islander | 0.3% |
| **Total** | **100.0%** |  |  |  |  | Other | 3.9% |
|  |  |  |  |  |  | White | 58.8% |
|  |  |  |  |  |  | **Total** | **100%** |
