# Supplementary material for: Pneumococcal colonization dynamics among young children with and without respiratory symptoms during the first year of the SARS-CoV-2 pandemic
Source: PLoS One. 2025 Jun 26;20(6):e0327046. doi: 10.1371/journal.pone.0327046 (PMC12200735; doi:10.1371/journal.pone.0327046)
Supplement: S1 File — S2 Appendix. Dates of community mitigation measures implemented in the Kansas City Metro Area. S1 Table. Procedure categories for which asymptomatic group required SARS-CoV-2 testing, by pneumococcal colonization status. S2 Table. Characteristics of asymptomatic participants. S3 Table. Complex chronic condition categories identified among patients in the asymptomatic and symptomatic groups. S1 Data. Minimal anonymized dataset. (ZIP) [file pone.0327046.s001.zip › S1 Table.docx]

**S1 Table**. Procedure categories for which asymptomatic group required SARS-CoV-2 testing, by pneumococcal colonization status

| **Procedure category** | **Colonized**  **(N=19)** | **Non-colonized**  **(N=133)** | **p value** |
| --- | --- | --- | --- |
| Hematology/Oncology | 0 (0.0%) | 2 (1.5%) | 0.59 |
| Orthopedics | 0 (0.0%) | 15 (11.3%) | 0.12 |
| Cardiology | 0 (0.0%) | 16 (12.0%) | 0.11 |
| Gastrointestinal | 4 (21.1%) | 24 (18.0%) | 0.75 |
| Neurology | 1 (5.3%) | 6 (4.5%) | 0.88 |
| Radiology | 4 (21.1%) | 21 (15.8%) | 0.56 |
| Urology/Nephrology | 2 (10.5%) | 12 (9.0%) | 0.83 |
| Dental | 2 (10.5%) | 8 (6.0%) | 0.46 |
| Ear, nose and throat | 4 (21.1%) | 19 (14.3%) | 0.44 |
| Ophthalmology | 2 (10.5%) | 10 (7.5%) | 0.65 |
