# Supplementary material for: Pneumococcal colonization dynamics among young children with and without respiratory symptoms during the first year of the SARS-CoV-2 pandemic
Source: PLoS One. 2025 Jun 26;20(6):e0327046. doi: 10.1371/journal.pone.0327046 (PMC12200735; doi:10.1371/journal.pone.0327046)
Supplement: S1 File — S2 Appendix. Dates of community mitigation measures implemented in the Kansas City Metro Area. S1 Table. Procedure categories for which asymptomatic group required SARS-CoV-2 testing, by pneumococcal colonization status. S2 Table. Characteristics of asymptomatic participants. S3 Table. Complex chronic condition categories identified among patients in the asymptomatic and symptomatic groups. S1 Data. Minimal anonymized dataset. (ZIP) [file pone.0327046.s001.zip › S2 Appendix.docx]

**S2 Appendix**. Dates of community mitigation measures implemented in the Kansas City Metro Area

| Date | Intervention |
| --- | --- |
| 3/16/20 | Schools closure |
| 3/24/20 – 5/5/20 | Stay-at-home order |
| 5/6/20 | Reopening Phase 1   - Residents should not gather in groups greater than 10 people - Entertainment venues, gyms, outdoor playgrounds, and sport courts must remain closed - Retail establishments, personal services such as salons, and dine-in restaurants may open but must limit the number of people |
| June 2020 | Reopening Phase 2, varied by county  In general, businesses percentage capacity limits are eliminated (except for taverns and bars, and large entertainment indoor facilities).  Child care services are allowed with certain restrictions. |
| 6/30/20 | Mask mandate: facemask to be worn in public spaces |
| Mid-August 2020 | Few schools offered in-person attendance. This gradually increased towards the end of this study and varied by school district.  By April 2021, all schools offered full-time, in-person attendance. |
