# Supplementary material for: Pneumococcal colonization dynamics among young children with and without respiratory symptoms during the first year of the SARS-CoV-2 pandemic
Source: PLoS One. 2025 Jun 26;20(6):e0327046. doi: 10.1371/journal.pone.0327046 (PMC12200735; doi:10.1371/journal.pone.0327046)
Supplement: S1 File — S2 Appendix. Dates of community mitigation measures implemented in the Kansas City Metro Area. S1 Table. Procedure categories for which asymptomatic group required SARS-CoV-2 testing, by pneumococcal colonization status. S2 Table. Characteristics of asymptomatic participants. S3 Table. Complex chronic condition categories identified among patients in the asymptomatic and symptomatic groups. S1 Data. Minimal anonymized dataset. (ZIP) [file pone.0327046.s001.zip › S2 Table.docx]

**S2 Table.** Characteristics of asymptomatic participants

|  | Exposed to SARS-CoV-2 (N=33) | Pre-procedure (N=152) | p value |
| --- | --- | --- | --- |
| Age < 2 years | 7 (21.2%) | 86 (56.6%) | 0.0002 |
| Male | 13 (39.4%) | 95 (62.5%) | 0.01 |
| Complex chronic conditions | 2 (6.1%) | 73 (48%) | <0.00001 |
| Smoking exposure | 6/28 (21.4%) | 18/150 (12%) | 0.2 |
| ≥ 3 doses of PCV13 | 27/31 (87.1%) | 93/137 (67.9%) | 0.03 |
| Positive SARS-CoV-2 PCR test | 13 (39.4%) | 20 (13.2%) | 0.0004 |
| Pneumococcal colonization | 6 (18.2%) | 19 (12.5%) | 0.4 |
