# Supplementary material for: Pneumococcal colonization dynamics among young children with and without respiratory symptoms during the first year of the SARS-CoV-2 pandemic
Source: PLoS One. 2025 Jun 26;20(6):e0327046. doi: 10.1371/journal.pone.0327046 (PMC12200735; doi:10.1371/journal.pone.0327046)
Supplement: S1 File — S2 Appendix. Dates of community mitigation measures implemented in the Kansas City Metro Area. S1 Table. Procedure categories for which asymptomatic group required SARS-CoV-2 testing, by pneumococcal colonization status. S2 Table. Characteristics of asymptomatic participants. S3 Table. Complex chronic condition categories identified among patients in the asymptomatic and symptomatic groups. S1 Data. Minimal anonymized dataset. (ZIP) [file pone.0327046.s001.zip › S3 Table.docx]

**S3 Table**. Complex chronic condition categories identified among patients in the asymptomatic and symptomatic groups.

| **Complex chronic condition categories** | **Asymptomatic (N=75)** | **Symptomatic (N=30)** | **p value** |
| --- | --- | --- | --- |
| Congenital or genetic defect | 8 (10.7%) | 1 (3.3%) | 0.23 |
| Cardiovascular | 25 (33.3%) | 13 (43.3%) | 0.34 |
| Gastrointestinal | 26 (34.7%) | 5 (16.7%) | 0.07 |
| Hematologic or immunologic | 15 (20.0%) | 9 (30.0%) | 0.27 |
| Malignancy | 10 (13.3%) | 6 (20.0%) | 0.39 |
| Metabolic | 5 (6.7%) | 3 (10.0%) | 0.56 |
| Premature and neonatal | 6 (8.0%) | 1 (3.3%) | 0.39 |
| Neurologic and neuromuscular | 20 (26.7%) | 8 (26.7%) | 1 |
| Renal or urologic | 21 (28.0%) | 7 (23.3%) | 0.63 |
| Respiratory | 27 (36.0%) | 9 (30.0%) | 0.56 |
| Technology dependency | 31 (41.3%) | 7 (23.3%) | 0.08 |
| Transplant | 4 (5.3%) | 1 (3.3%) | 0.66 |
